# Temporal trends in diagnostic work-up, treatment, and mortality in locally advanced prostate cancer in 2016–2024: nationwide, population-based study in Sweden

**DOI:** 10.2340/ao.v65.45593

**Published:** 2026-04-15

**Authors:** Armando Galdieri, Hans Garmo, Rolf Gedeborg, Mats Ahlberg, Andri Wilberg Orrason, Eugenio Ventimiglia, Pär Stattin, Marcus Westerberg

**Affiliations:** a Department of Surgical Sciences, Uppsala University, Uppsala, Sweden; b Unit of Urology/Division of Oncology, Gianfranco Soldera Prostate Cancer Lab, IRCCS San Raffaele Scientific Institute, Milan, Italy; c Vita-Salute San Raffaele University, Milan, Italy

**Keywords:** Locally advanced prostate cancer, treatment, mortality, diagnosis, staging

## Abstract

**Background and purpose:**

We aimed to describe temporal changes in diagnostic work-up, treatment, and prostate cancer (PCa) mortality in locally advanced PCa in 2016–2024 in Sweden.

**Patient/material and methods:**

Men registered in the National Prostate Cancer Register of Sweden in 2016–2024 with locally advanced PCa; clinical T stage 3–4, no distant metastases, and prostate-specific antigen < 100 ng/ml were included. We computed the proportion of use of prostate magnetic resonance imaging (MRI) before biopsy, radical prostatectomy, radical radiotherapy, androgen deprivation therapy, and abiraterone, and described the trend in PCa mortality across three calendar periods.

**Results:**

During the 9-year study period 7,484 men with locally advanced PCa were identified. Use of MRI before biopsy increased from 3% in 2016 to 73% in 2024. Concomitantly, radical treatment increased from 35% to 48%, entirely due to increased use of radiotherapy. Abiraterone was not used before 2022 but 31% received this treatment in 2024. The 3-year PCa mortality decreased from 8% (95% confidence interval [CI]: 7–9) in 2016–2018 to 6% (95% CI: 4–8) in 2022–2024.

**Interpretation:**

In this nationwide, population-based study of men with locally advanced PCa, the use of MRI before biopsy, radical radiotherapy, and treatment with abiraterone increased over time. These changes coincided with a modest decrease in 3-year PCa mortality.

## Introduction

There have been major changes in diagnostic work-up of prostate cancer (PCa) in the last decade. The introduction of prostate magnetic resonance imaging (MRI) prior to biopsy has increased the diagnostic accuracy for clinically significant PCa while prostate-specific membrane antigen positron emission tomography (PSMA-PET) has improved the detection of nodal and distal metastases [1–3].

In parallel, the use of radical radiotherapy (RT) with neoadjuvant and adjuvant androgen deprivation therapy (ADT) for men diagnosed with locally advanced PCa has increased [4–9]. The androgen receptor pathway inhibitor (ARPI) abiraterone has been shown to prolong survival for men with locally advanced PCa. Therefore, abiraterone is recommended since 2022 in American, European and Swedish guidelines for men with PCa with additional risk factors for more aggressive disease [10–15].

It has previously been shown that the use of radical treatment in men with locally advanced PCa increased between 2000 and 2016 in Sweden, with a concomitant decrease in 5-year prostate cancer mortality [16], but the impact on clinical practice of these recent recommendations and the concomitant trend in PCa mortality is unknown.

The aim of this study was to describe the temporal changes in diagnostic work-up, treatment, and PCa mortality in men diagnosed with locally advanced PCa in Sweden in 2016–2024.

## Patients/material and methods

### Data sources

The National Prostate Cancer Register (NPCR) of Sweden captures 98% of all men diagnosed with PCa in Sweden since 1998, compared with the Cancer Register, to which reporting is mandated by law. The NPCR contains comprehensive data on cancer characteristics at diagnosis, as well as detailed information on diagnostic work-up and primary treatment. The primary aim of NPCR is to assess adherence to national guidelines and thereby ensure high-quality care of men with PCa [17–19].

In Prostate Cancer data Base Sweden (PCBase), NPCR has been linked to multiple national health care registers and demographic databases by use of the unique Swedish personal identity number [20]. These linkages include the Patient Register, the Cancer Register, the Prescribed Drug Register, the Total Population Register, and the Cause of Death Register. In 2025, data from healthcare information systems from all 21 regions in Sweden on prostate specific antigen (PSA), prostate biopsies, and prostate MRI, and hospital administered treatments were added to PCBase to create PCBase Xtend [21].

This project was approved by the Swedish Research Ethics Authority.

### Variables extracted

Data on age at diagnosis, date of diagnosis, MRI before diagnostic biopsy, PSMA-PET use imaging of lymph nodes, PSA values, Gleason score (GS), clinical TNM stage, date of radical treatment (radical prostatectomy or radical radiotherapy), and fraction and total dose of radiotherapy, was extracted from NPCR. The start of ADT (Anatomical Therapeutic Chemical [ATC] codes: L02BB03 [bicalutamide], L02BX02 [degarelix], L02BX04 [relugolix]) or abiraterone (ATC code L02BX03) were defined as the earliest date of a dispensed prescription for these drugs in the Prescribed Drug Register or a recorded administration by healthcare staff. Health-adjusted life expectancy at diagnosis was estimated based on age at diagnosis and comorbidity burden measured with a Multidimensional Diagnosis-based Comorbidity index (MDCI) and a Drug Comorbidity Index (DCI) [22–24]. Date of emigration was extracted from the Total Population Register. Date and cause of death were extracted from the Cause of Death Register.

### Study population

All men diagnosed with locally advanced PCa between 2016 and 2024 and registered in NPCR were included. Locally advanced PCa was defined as clinical T stage 3–4, no evidence of metastatic disease (M0), any N stage, any GS, and PSA < 100 ng/ml.

### Outcomes

We assessed the use of prostate MRI before biopsy, PSMA-PET and the use of imaging for nodal staging in the diagnostic work-up. Treatment with radical prostatectomy, RT, ADT, and abiraterone was assessed up to 6 months after diagnosis. We also assessed death from PCa and overall mortality.

### Follow-up

Follow-up started at date of diagnosis and ended 31 December 2024, date of emigration, or date of death, whichever occurred first.

### Statistical analysis

We described the annual use of MRI before biopsy, PSMA-PET, nodal imaging, type of radical treatment received, ADT, and abiraterone using crude proportions. Cumulative incidence of PCa mortality up to 5 years after diagnosis was described while considering death from other causes as a competing risk. Mortality data were presented with 95% confidence intervals (CI).

Analyses were stratified in three calendar periods (2016–2018, 2019–2021, and 2022–2024) and further stratified by age at diagnosis (< 70, 70–79, > 79 years). We assessed the use of abiraterone separately in men with one STAMPEDE risk factor, that is, stage T3-4 that was an inclusion criteria to our study group, and in men with two or more STAMPEDE risk factors (T3-4 + at least one of: N+, GS 8–10 or PSA > 40 ng/ml) [15].

Analyses of treatment were also stratified according to life expectancy (< 5, 5–10, and > 10 years).

All analyses were performed using R: A language and environment for statistical computing (R Foundation for Statistical Computing, Vienna, Austria), version 4.5.1.

## Results

### Baseline characteristics

There were 7,484 men diagnosed with locally advanced prostate cancer in 2016–2024. Median PSA value at diagnosis showed a slight decrease over time and there was modest shift towards higher GS over time (Table 1).

**Table 1:** Baseline characteristics of men diagnosed with locally advanced prostate cancer between 2016 and 2024 and registered in the National Prostate Cancer Register of Sweden.

	2016–2018		2019–2021		2022–2024	
	*N*	%	*N*	%	*N*	%
	2,575	100	2,292	100	2,609	100
**Age at biopsy** (years)						
Median (IQR)	75 (69–81)		76 (70–81)		76 (69–80)	
< 70	679	26	554	24	666	25
70–79	1,106	43	1,020	45	1,177	45
> 79	790	31	721	31	771	29
**Life expectancy** (years)						
Median (IQR)	11.2 (7.4–15.5)		11.0 (7.1–15.3)		11.1 (7.8–15.3)	
< 5	283	11	265	12	238	9.1
5–10	792	31	727	32	863	33
> 10	1,500	58	1,303	57	1,513	58
**PSA** (ng/ml)						
Median (IQR)	19 (9–37)		18 (9–38)		17 (8–35)	
< 5	201	7.8	174	7.6	249	9.5
5–9.9	511	20	496	22	576	22
10–19.9	612	24	569	25	632	24
20–49.9	814	32	693	30	762	29
50–99.9	437	17	363	16	395	15
**MRI before biopsy**	97	4	751	33	1,709	65
**Nodal imaging use**	1,311	51	1,198	52	1,448	55
**Gleason score**						
6	145	5.9	88	4.1	71	2.8
3+4	487	20	372	17	454	18
4+3	513	21	469	22	564	22
8	432	17	344	16	422	17
9–10	898	36	881	41	994	40
Missing	100		138		105	
**T stage**						
3	2,418	94	2,138	93	2,453	94
4	157	6.1	157	6.8	161	6.2
**N stage**						
0	1,023	40	945	41	1,126	43
1	288	11	253	11	321	12
x	1,264	49	1,097	48	1,175	45

IQR: interquartile range.

### Trends in diagnostic work-up

**Figure 1:**
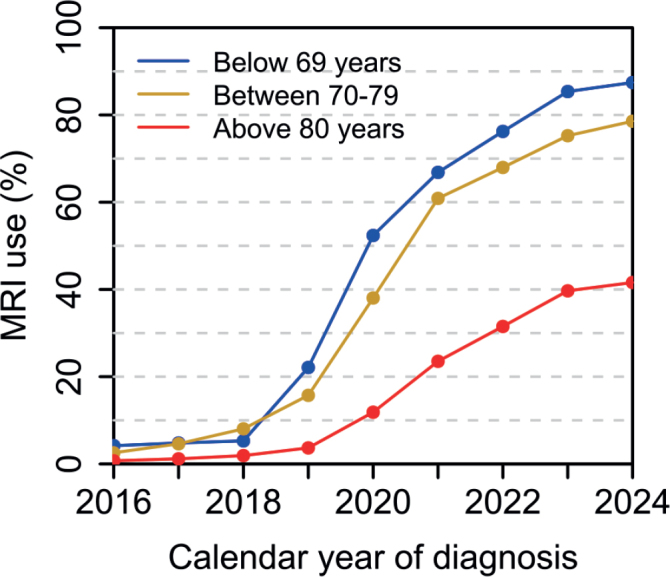
Use of prostate magnetic resonance imaging before biopsy in all men with locally advanced prostate cancer.

Between 2016 and 2024, the use of MRI before biopsy increased from 3% to 73% and use of imaging of lymph nodes increased from 48% to 55%. The increase in MRI use was largest in men under 70 years, rising from 4% to 89% (Figure 1), while lymph node imaging showed the largest increase in men over 79 years (from 10% to 42%). PSMA-PET was not used before 2020 and was 17% in 2024, with the highest use in men aged below 70 (26%)*.*

### Treatment trends

**Figure 2:**
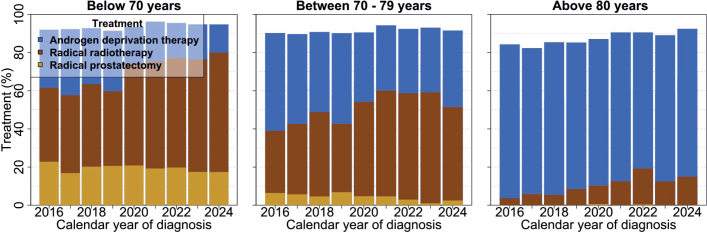
Use of radical treatments and androgen deprivation therapy within 6 months from date of diagnosis stratified by age. The white area represents men who did not receive any treatment.

Between 2016 and 2024, radical treatment within 6 months from diagnosis increased from 35% to 48%, primarily due to an increase use of radiotherapy, which increased from 26% to 43% (Figure 2). A majority (63%) of men with life expectancy above 10 years underwent radical treatment while few men (4%) with life expectancy below 5 years underwent radical treatment (Supplementary Figure 1).

Among men who received radiotherapy the use of conventional radiotherapy decreased whereas use of hypofractionated radiotherapy increased almost two-fold, from 26% to 43%, and use of ultra-hypofractionated radiotherapy increased from no use to 15% (Supplementary Figure 2). Lymph nodes were included in the RT field in 4% of men who underwent RT in 2016 and 16% of men in 2024.

Among men who received radical prostatectomy (RP) the use of robotic-assisted laparoscopic prostatectomy (RALP) increased from 79% to 100%. No laparoscopic RP or open RP was performed in 2024. Use of lymph node dissection during RP decreased from 64% to 24%.

The use of ADT decreased from 54% in 2016 to 45% in 2024 (Figure 2). Neoadjuvant and adjuvant ADT with RT increased from 95% to 99%.

**Figure 3:**
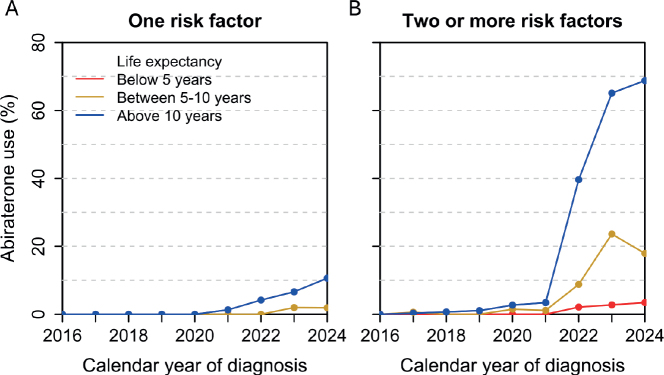
Use of abiraterone within 6 months from date of diagnosis according to life expectancy. (A) Men with one STAMPEDE risk factor (T3-4 stage, which was inclusion criteria to the study group). (B) Two or more STAMPEDE risk factors (T3-4 + at least one of: N+, Gleason score 8–10 or PSA > 40 ng/ml).

Use of abiraterone increased from no use before 2022 to 31% in 2024 (Figure 3). This increase occurred almost exclusively in men with two or more risk factors, out of which 44% received abiraterone. Among these, 69% of men with life expectancy > 10 years received abiraterone.

### Mortality trends

**Figure 4:**
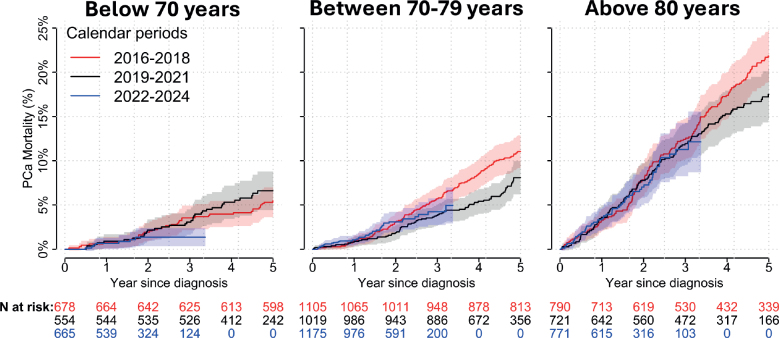
Cumulative incidence proportion of up to 5-year prostate cancer mortality stratified by age.

The 3-year PCa mortality risk was 8% (95% CI: 7–9) in 2016–2018 and 6% (95% CI: 4–8) in 2022–2024 and the 3-year overall mortality risk was 18% in 2016–2018 and 16% in 2022–2024. In the subgroup of men below age 70, the 3-year PCa mortality risk was 4% (95% CI: 2–5) in 2016–2018 and 1% (95% CI: 0.3–2) in 2022–2024 (Figure 4). In this group the 5-year PCa mortality was 5.7% (95% CI: 4–7) in 2016–2018 and 6.5% (95% CI: 4–9) in 2019–2021. Other age-groups followed the overall trend.

## Discussion and conclusion

### Summary of findings

In this nationwide, population-based study of men with locally advanced PCa diagnosed in 2016–2024, use of MRI before biopsy increased drastically. Use of radical radiotherapy increased slightly and use of abiraterone increased from no use before 2022 to one third of men with an even higher use in men with multiple risk factors and long life expectancy. These trends were parallelled by a modest decrease in 3-year prostate cancer mortality and overall mortality.

### Interpretation and previous studies

The decreased PSA levels at diagnosis and increase of high-grade disease (GS > 8) are consistent with the previously reported trends [16]. During the study period, prostate MRI use before biopsy increased as expected following the 2019 guideline recommendations, and PSMA-PET use started in 2020 after its inclusion in major guidelines [1, 3, 10, 11, 25].

The trends in the use of radical treatment previously reported from 2000 to 2016 continued in this study period [15]. The increase in radical RT use was driven by more hypofractionated and ultra-hypofractionated radiotherapy. This change follows the 2019 guideline recommendations based on the HYPO-RT-PC trial [13, 26, 27]. Use of radical RT was the highest in men with life expectancy > 10 years, in line with recommendations in current guidelines [13, 24]. The results of the ongoing SPCG-15 trial that compares radical prostatectomy with radiotherapy in men with locally advanced PCa, may further influence future guidelines and clinical practice [28].

The STAMPEDE trial demonstrated improved survival for the addition of abiraterone to ADT compared with standard of care (76 vs. 45 months) in men with high risk PCa, after a median follow-up of 7 years. These findings led to the incorporation of abiraterone into Swedish guidelines in 2022 [10–14, 29].

By 2024, nearly half of men with two or more risk factors received abiraterone. The follow-up in this study was short, in particular for men diagnosed in the latest calendar period (2022–2024), and longer follow-up is needed to fully assess how the uptake of abiraterone affects PCa mortality.

The modest decrease in 3-year PCa mortality in 2016–2024 that we observed in all men diagnosed with locally advanced PCa in Sweden, not just men who received recommended treatment, is a robust finding that corroborates improved outcome for men with locally advanced PCa. This finding is a continuation of the improvement that we observed in our previous study [16], and is in accordance with the decline in PCa mortality in all stages in the Nordic countries [30]. The higher 5-year PCa mortality in men aged below 70 years in 2019–2021 compared to 2016–2018 is counterintuitive. Further investigation is warranted to understand the cause of this increase.

### Strengths and limitations

A strength of this study is that virtually all men with locally advanced PCa in Sweden could be included [17] with comprehensive data on cancer characteristics, primary treatment, use of ADT, and follow-up was virtually complete. The recency of the data is another strength, the last date of follow-up was December 2024. The study also has some limitations. The follow-up time for men who were recently diagnosed was short, so we did not capture the full effect of improvements in diagnostics and treatment. Stage migration is likely a contributing factor to the observed decrease in mortality since improved imaging can detect small metastases [3, 31, 32, 33]. Finally, we were unable to fully assess the cost-benefit ratio of more radical treatment, which ideally would have been done, given that radical radiotherapy with neoadjuvant and adjuvant ADT has well-known long-term side effects.

## Conclusions

In this nationwide, population-based study of men with locally advanced PCa we observed, in accordance with changes in national guidelines, increased use of MRI before biopsy, radical radiotherapy, and abiraterone over time. In parallel, there was a continued modest decrease in prostate cancer mortality.

## Supplementary Material





## Data Availability

Data used in this study were extracted from the Prostate Cancer data Base Sweden (PCBase), which is based on the National Prostate Cancer Register (NPCR) of Sweden and linkage to several national health-data registers. The data cannot be shared publicly because the individual-level data contain potentially identifying and sensitive patient information and cannot be published due to legislation and ethical approval (https://etikprovningsmyndigheten.se). Use of the data from national health-data registers is further restricted by the Swedish Board of Health and Welfare (https://www.socialstyrelsen.se/en/) and Statistics Sweden (https://www.scb.se/en/), which are Government Agencies providing access to the linked healthcare registers. The data will be shared on reasonable request in an application made to any of the steering groups of NPCR and PCBase (contact npcr@npcr.se). To request data or analytic code from this study, contact the corresponding author. For detailed information, please see www.npcr.se/in-english, where registration forms, manuals, and annual reports from NPCR are available alongside a full list of publications from PCBase.
