# Supplementary material for: Temporal trends in diagnostic work-up, treatment, and mortality in locally advanced prostate cancer in 2016–2024: nationwide, population-based study in Sweden
Source: Acta Oncol. 2026 Apr 15;65:AO-65-45593. doi: 10.2340/ao.v65.45593 (PMC13090860; doi:10.2340/ao.v65.45593)

Supplementary material has been published as submitted. It has not been copyedited, or typeset by Acta Oncologica

Supplementary Figure 1: Use of radical treatment and androgen deprivation therapy, within six months from diagnosis, according to life expectancy. The white area represents patients who did not receive any active treatment.

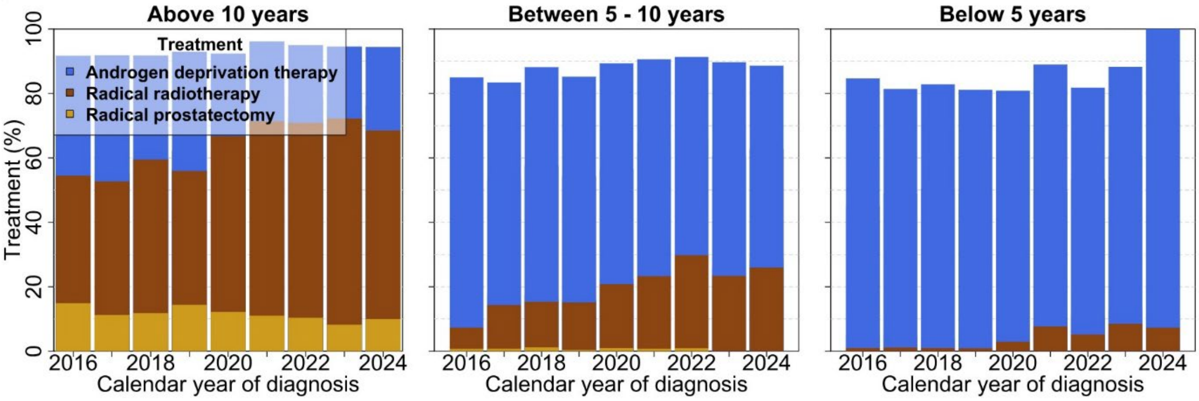

Supplement: Supplementary file 1 [file AO-65-45593-s1.pdf]
