# Supplementary material for: Temporal trends in diagnostic work-up, treatment, and mortality in locally advanced prostate cancer in 2016–2024: nationwide, population-based study in Sweden
Source: Acta Oncol. 2026 Apr 15;65:AO-65-45593. doi: 10.2340/ao.v65.45593 (PMC13090860; doi:10.2340/ao.v65.45593)

Supplementary material has been published as submitted. It has not been copyedited, or typeset by Acta Oncologica

Supplementary Figure 2: Type of radiotherapy in men with locally advanced prostate cancer that received radiotherapy within six months from diagnosis.

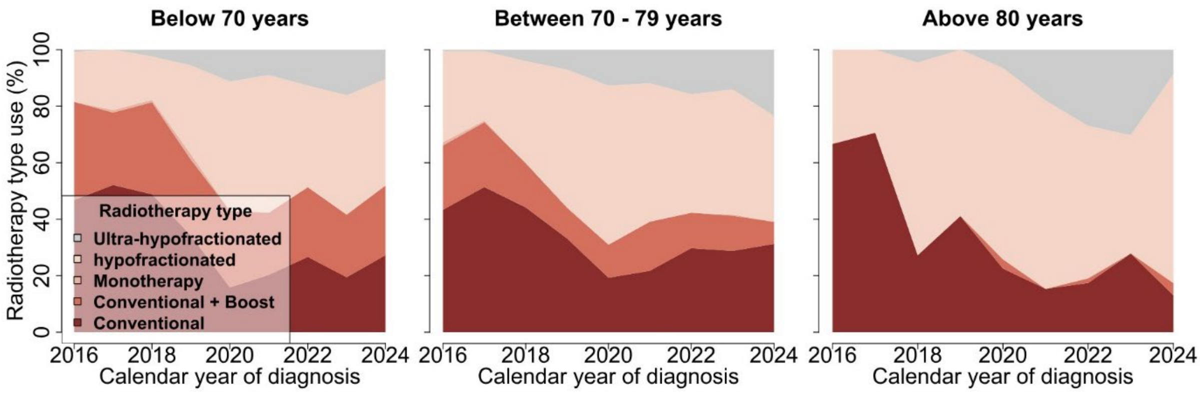

Supplement: Supplementary file 2 [file AO-65-45593-s2.pdf]
